# Alpha rhythm slowing in a modified thalamo-cortico-thalamic model related with Alzheimer’s disease

**DOI:** 10.1371/journal.pone.0229950

**Published:** 2020-03-12

**Authors:** XiaoYuan Li, XiaoLi Yang, ZhongKui Sun

**Affiliations:** 1 College of Mathematics and Information Science, Shaanxi Normal University, Xi’an, PR China; 2 Department of Applied Mathematics, Northwestern Polytechnical University, Xi’an, PR China; Radboud Universiteit, NETHERLANDS

## Abstract

A decrease in alpha band power is defined as a hallmark of electroencephalogram (EEG) in Alzheimer’s disease (AD). This study devotes to understanding the neuronal correlates of alpha rhythm slowing associated with AD from the view of neurocomputation. Firstly, a modified computational model of thalamo-cortico-thalamic (TCT) circuitry is constructed by incorporating two important biologically plausible ingredients. One is the disinhibition property between different inhibitory interneurons in the cortical module. The other is the full relay function of thalamic relay nucleus (TCR) to the cortical module. Then, by decreasing synaptic connectivity parameters to mimic the neuropathological condition of synapse loss in AD, the correlation between neuronal synaptic behavior and abnormal alpha rhythm is simulated by means of power spectral analysis. The results indicate that these decreases of synaptic activity, i.e., not only the excitatory synaptic connections from TCR to fast inhibitory interneurons *C*_*fte*_ and from excitatory interneurons to pyramidal neurons *C*_*pxe*_ but also the inhibitory synaptic connections from fast inhibitory interneurons to slow inhibitory interneurons *C*_*lfi*_ and from inhibitory interneurons to TCR *C*_*tii*_, can significantly diminish the peak power density over the alpha band of the thalamic output, which implies that there is a slowing of alpha band. Furthermore, the underlying mechanism behind the alpha rhythmic changes is analyzed using nonlinear dynamical technique. The results reveal that decreases of *C*_*fte*_, *C*_*pxe*_, *C*_*lfi*_ and *C*_*tii*_ can make the thalamic module transfer from a limit cycle mode to a point attractor mode, which may lead to the alpha rhythm slowing in the modified TCT model. We expect this work can be helpful in identifying early biomarkers of AD’s EEG and understanding potential pathogenesis of AD.

## Introduction

As one of the most common forms of dementia, Alzheimer’s disease (AD) primarily affects central system of the brain and causes neuronal degenerative changes. The statistics shows that about 50%-60% of patients with dementia are associated with AD. AD is quite pervasive among elderly population around the world. As the age of the elderly steadily grows, the clinical symptoms such as cognitive decline and blurred expression continue to emerge, which makes Alzheimer’s patients not competent to cope with their daily life and social activities. Thus, Alzheimer’s disease especially its discovery and diagnosis has aroused widespread attention recently. Note that the early symptoms of AD are related with normal aging [1]. The pathological features of AD become apparent only when the brain is irreparably destroyed [2]. These facts make it hard to distinguish the early clinical signs of AD from other age-related mental dementia. On the other hand, though drug therapy by acting medicines on different neurotransmitter systems can delay the development of AD in clinical practice [3], it is usually carried out at the intermediate stage of AD when neurons in cognitive regions are irreversibly damaged [4]. In addition, the efficacy of drug treatment is poor and its expenses are vast. Therefore, one challenge of AD is searching for biomarkers which facilitate to detect this disease before it causes serious deterioration in brain.

Among AD’s biomarkers, electroencephalogram (EEG) abnormality is one of the potential indicators. Owing to the EEG recording system is inexpensive and non-invasive, it has become a more popular and feasible tool for finding symbols in various kinds of pathological and neurological diseases [5]. Since Hans Berger firstly observed clinical EEG sequences in an AD patient [6], the EEG of AD has been extensively studied with conventional visual analysis [7–9]. Recent research has reported that rhythm slowing is a significant characteristic of EEG abnormality for AD patients [7–10]. A decrease in posterior rhythm and an increase in diffuse slow activity have also been discovered in AD patients’ EEG by visual analysis [7, 8]. In addition, Refs. [9, 10] have revealed that EEG of AD patients exhibits a reduction or absence of alpha rhythm.

Because the visual analysis of EEG is subjective and somewhat inaccurate, some supplementary techniques have been introduced to better analyze and extract the characteristic of EEG. Spectral analysis of EEG is a quantitative method for studying AD related features [11]. It has been demonstrated that a decrease in power over the alpha band of EEG is identified as a hallmark of AD [12, 13]. For example, Basar et al. have reported the remarkable difference between AD patients who are treated with cholinesterase inhibitors and those who are not treated is the alpha frequency band [12]. By spectral analysis of EEG in patients with moderate to severe Alzheimer’s disease, Soininen et al. have suggested that a distinct feature of EEG in AD patients is a reduction of alpha band power, i.e., the slowing of alpha band [13]. The technique of nonlinear dynamical analysis has also been employed to explore the EEG abnormality in AD. For example, the relationship between EEG coherence and correlation dimension has been revealed by using nonlinear analysis [14]. Hornero et al. have discussed the effectiveness of nonlinear dynamical method in analyzing EEG and magnetoencephalography (MEG) of AD patients, and believed that this method can contribute to diagnose AD [15]. Furthermore, an advanced method combing nonlinear dynamical analysis and spectral analysis has been proposed to investigate rhythm changes of EEG [16], interested readers please prefer to Refs. [17, 18].

To better understand the neuronal correlates of abnormal EEG, much attention has been gradually shifted to computational model associated with neurological or psychiatric disorders [19–22]. For AD, several neuronal models related with the hippocampal region, one of the major sources of low frequency theta oscillation, have been established to study the hippocampal theta rhythm together with memory loss [18]. Note that Braak et al. have pointed out that one remarkable change of brain structures in AD patients is cortical atrophy [23]. Neuroimaging detection on AD patients and normal people has demonstrated that the atrophy of thalamic structure occurs during the development of AD [24]. It is also known that the resting state oscillatory activity observed in EEG is mainly affected by neuron populations in the thalamic and cortical tissues [25–27]. Thus, some neuronal computational models including cortex and thalamus circuits have already emerged to explore abnormal brain rhythmic activity. For example, based on biophysical and histological data coming from Tombol’s Golgi experiment on the thalamus of adult cat, Lopes da Silva et al. have established a classical alpha rhythm computational model, which includes two interconnected thalamus neuron populations by means of negative feedback mimicking alpha rhythm [28]. Subsequently, Freeman has constructed a similar model to detect the dynamical behavior of olfactory cortex [29]. This model is further modified and extensively investigated [30–33]. Moran et al. have incorporated an inhibitory self-loop among inhibitory interneurons to simulate spectral density of EEG and MEG recordings [31, 32]. With the inclusion of the self-connections between *GABA*_*A*,*fast*_ interneurons, Ursino et al. have developed a modified neural mass model to simulate a variety of brain oscillations during wakefulness, such as alpha, beta and gamma rhythms. They have also analyzed the reciprocal influences between different rhythms in a “system of rhythms” by connecting two or three cortical regions with different topologies of long range connection [34]. Cona et al. have established a thalamocortical computational model by introducing thalamo-cortical neuron population and thalamo-reticular neuron population in the Ursino model, and mimicked the different patterns of rhythmic activity in cortical and thalamic neurons [35]. In addition, Bhattacharya et al. also have employed the computational model of thalamocortical circuit to explain the possible neuronal population behavior linked with abnormal alpha rhythmic activity in AD, in which they found that inhibitory synaptic activity can lead to slowing of the alpha rhythm [36]. Subsequently, Bhattacharya et al. have constructed an improved thalamocortical model by modifying the structure of a single neuronal population, and they have concluded that the inhibitory synaptic connection is directly related to alpha rhythm slowing [17]. Recently, a more biologically plausible TCT model has been proposed to investigate the underlying causes of abnormal brain rhythm in AD condition [37].

Note that using advanced techniques for tissue slicing and morphological reconstruction, Jiang et al. have found that there are at least 15 types of inhibitory neurons in the human brain. These inhibitory neurons are roughly subdivided into three major groups, one of which preferentially inhibits the electroactivity of self-type inhibitory neurons and also inhibits the electroactivity of excitatory neurons [38]. Meanwhile, by combining optogenetic activation with single cell recordings, Pi et al. have detected a unique mode of inhibitory control which may be provided by inhibitory neurons that specifically suppress the firing of other inhibitory neurons [39]. This means that there has been a basic disinhibitory circuit in the mammalian cerebral cortex, and the disinhibition property indicates there are mutual effects between different inhibitory neurons. In addition, the thalamus is the brain’s major center for processing sensory information. It is composed of 15 relay thalamo-cortical nuclei (TCN) and the TRN [35], in which these TCNs contain TCR cell population. TCNs transmit sensory information from the periphery to the cortex, in particularly the TCR sends excitatory and AMPA-mediated synapses to cortical areas and to the TRN, performing a function rather like a junction or relay station. For simplicity, the above alpha rhythm models and the modified versions have ignored the interactions between different inhibitory neurons. Also, they have not considered the projection of thalamic relay nucleus in the thalamic module to the cerebral cortex fully. Based on these findings, this study by incorporating these two important biologically plausible ingredients, i.e., the disinhibition property between different inhibitory interneurons in the cortical module as well as the full relay function of thalamus to the cortical module, attempts to construct a modified neuronal model of TCT circuit on the basis of work [37]. We believe that this modified TCT model is related with AD, which is a good candidate to understand the thalamo-cortical-thalamic neuronal mechanism associated with alpha rhythmic slowing observed in AD.

In this computational model, by decreasing synaptic connectivity parameters to mimic the neuropathological condition of synapse loss in AD, we simulate the correlation between neuronal synaptic behavior and abnormal alpha rhythm in AD by means of power spectral analysis. Furthermore, the underlying mechanism behind the alpha rhythmic changes is analyzed using nonlinear dynamical technique. The structure of this work is as follows. Firstly, the modified TCT model and the preliminary of numerical simulation are presented. Then the influence of excitatory and inhibitory synaptic activity on the power spectra within alpha band is deeply explored. Finally, we make a brief summary and discussion of this work.

## Model presentation and preliminary

**Fig 1:**
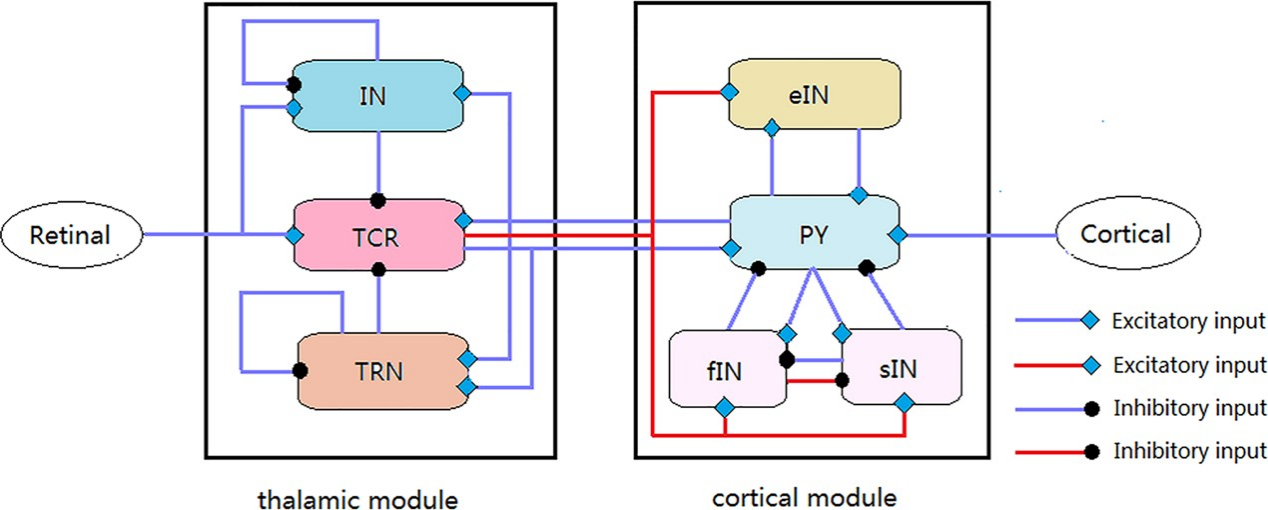
Schematic diagram of the modified TCT model presented in this work. The synaptic structures of the thalamic and the cortical module are based on Ref. [40] and Refs. [33, 41], respectively. Blue lines with arrow heads and round heads denote the excitatory and inhibitory synaptic projections existing in the original TCT model, respectively. Red lines with arrow heads and round heads indicate the excitatory and inhibitory synaptic projections newly introduced in the modified TCT model, respectively.

**Fig 2:**
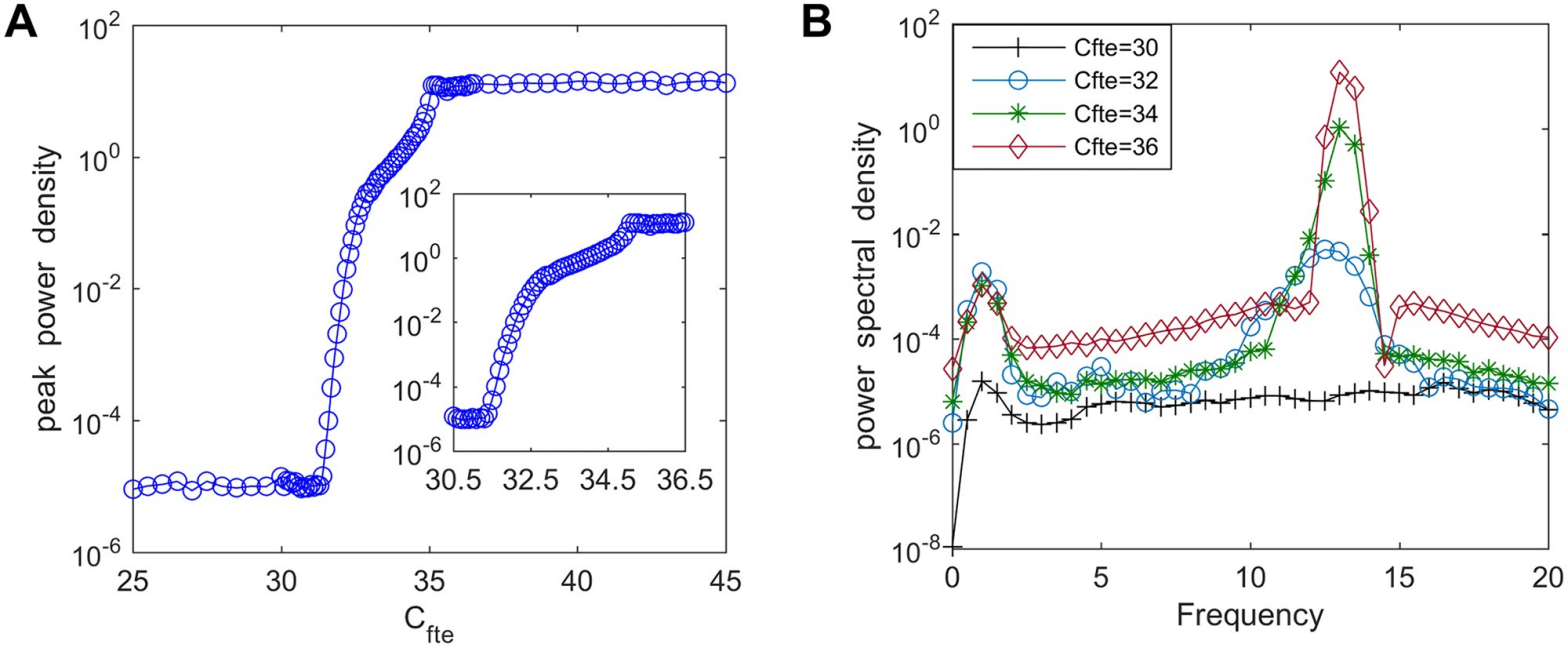
The peak power density and the corresponding power spectral density for varying values of *C*_*fte*_. (A) The peak power density within the alpha band of the thalamic output. (B) The corresponding power spectral density for different connectivity parameters such as *C*_*fte*_ = 30, 32, 34, 36.

The present computational model constructed in this work can be considered as an improved version of the original TCT model [37]. Its organizational structure along with synaptic connectivity layout is displayed in the schematic diagram of Fig 1. As described in Ref. [37], the original TCT model includes two parts of the thalamic and the cortical module. The thalamic module comprises three neuron populations: thalamic relay nucleus (TCR), inhibitory interneurons (IN) and thalamic reticular nucleus (TRN). The cortical module consists of excitatory pyramidal neuron population (PY), excitatory interneuron population (eIN), slow inhibitory interneuron population (sIN) and fast inhibitory interneuron population (fIN). Within the thalamic module, both the IN and TRN neuron populations make inhibitory (GABAergic) synapses with the TCR neuron population, whereas the TCR neurons only send excitatory feedback to the TRN neurons. The IN and TRN neuron populations make inhibitory synapses on itself, respectively. Within the cortical module, the PY neuron population makes excitatory synapses with the other three neuron populations of eIN, sIN and fIN, respectively. Meanwhile, the two neuron populations of sIN and fIN send inhibitory feedback to the PY neuron population, and the eIN neuron population sends excitatory feedback to the PY neuron population. The sIN neuron population makes inhibitory synapses with the fIN neuron population. As for the synaptic connection between the thalamic and the cortical module, the excitatory projections on all the thalamic neuron populations from the PY neuron population are built. Conversely, only the excitatory projection on the PY neuron population from the relay nucleus is built to execute the TCR’s relay function. The other extrinsic source to the TCT model comes from the retinal and nearby cortical formation, i.e., the retinal population sends excitatory input to the IN and TCR neuron populations, and the PY neuron population receives excitatory afferent from nearby cortical regions to form cortico-cortical connection. As stated in the Introduction, the results in Refs. [38, 39] have implied that there exists a basic disinhibitory circuit in the mammalian cerebral cortex and there are mutual effects between different inhibitory neurons. On the same time, the key relay function of relay nucleus in the thalamus implies that the TCR neuron population can process afferent information and then transmit it to all the cortical populations. Thus, this present work has incorporated the following two improvements into the original TCT model: 1)Introducing inhibitory projection on the sIN neuron population from the fIN neuron population to construct disinhibitory circuit in the cortical module (indicated by red lines with round heads in Fig 1); 2) Introducing excitatory projections on the eIN, fIN and sIN neuron populations from the TCR neurons to achieve the full relay function of the thalamic relay nucleus (indicated by red lines with arrow heads in Fig 1). Then, the dynamical behavior for all neuron populations involved in this modified TCT model can be mathematically described by a series of first order differential equations, which are defined as follows:

**Retinal**:

$${\dot{x}}_{ret1}=x_{ret2}$$

$${\dot{x}}_{ret2}=\frac{H_{e}}{\tau_{e}}P_{1}(t)-\frac{2}{\tau_{e}}x_{ret2}-\frac{1}{{\tau_{e}}^{2}}x_{ret1}$$ (1)

**Cortico-cortical**:

$${\dot{x}}_{cc1}=x_{cc2}$$

$${\dot{x}}_{cc2}=\frac{H_{e}}{\tau_{e}}P_{2}(t)-\frac{2}{\tau_{e}}x_{cc2}-\frac{1}{{\tau_{e}}^{2}}x_{cc1}$$ (2)

**TCR**:

$${\dot{x}}_{tcr1}=x_{tcr2}$$

$${\dot{x}}_{tcr2}=\frac{H_{e}}{\tau_{e}}S(C_{tre}x_{ret1}+C_{tpe}x_{py1}-C_{tii}x_{in1}-C_{tni}x_{trn1})-\frac{2}{\tau_{e}}x_{tcr2}-\frac{1}{{\tau_{e}}^{2}}x_{tcr1}$$ (3)

**IN**:

$${\dot{x}}_{in1}=x_{in2}$$

$${\dot{x}}_{in2}=\frac{H_{i}}{\tau_{i}}S(C_{ire}x_{ret1}+C_{ipe}x_{py1}-C_{isi}x_{in1})-\frac{2}{\tau_{i}}x_{in2}-\frac{1}{{\tau_{i}}^{2}}x_{in1}$$ (4)

**TRN**:

$${\dot{x}}_{trn1}=x_{trn2}$$

$${\dot{x}}_{trn2}=\frac{H_{i}}{\tau_{i}}S(C_{nte}x_{tcr1}+C_{npe}x_{py1}-C_{nsi}x_{trn1})-\frac{2}{\tau_{i}}x_{trn2}-\frac{1}{{\tau_{i}}^{2}}x_{trn1}$$ (5)

**PY**:

$${\dot{x}}_{py1}=x_{py2}$$

$${\dot{x}}_{py2}=\frac{H_{e}}{\tau_{e}}S(C_{pce}x_{cc1}+C_{pte}x_{tcr1}+C_{pxe}x_{ein1}-C_{pli}x_{sin1}-C_{pfi}x_{fin1})-\frac{2}{\tau_{e}}x_{py2}-\frac{1}{{\tau_{e}}^{2}}x_{py1}$$ (6)

**eIN**:

$${\dot{x}}_{ein1}=x_{ein2}$$

$${\dot{x}}_{ein2}=\frac{H_{e}}{\tau_{e}}S(C_{xpe}x_{py1}+C_{xte}x_{tcr1})-\frac{2}{\tau_{e}}x_{ein2}-\frac{1}{{\tau_{e}}^{2}}x_{ein1}$$ (7)

**sIN**:

$${\dot{x}}_{sin1}=x_{sin2}$$

$${\dot{x}}_{sin2}=\frac{H_{il}}{\tau_{il}}S(C_{lpe}x_{py1}+C_{lte}x_{tcr1}-C_{lfi}x_{fin1})-\frac{2}{\tau_{il}}x_{sin2}-\frac{1}{{\tau_{il}}^{2}}x_{sin1}$$ (8)

**fIN**:

$${\dot{x}}_{fin1}=x_{fin2}$$

$${\dot{x}}_{fin2}=\frac{H_{if}}{\tau_{if}}S(C_{fpe}x_{py1}+C_{fte}x_{tcr1}-C_{fli}x_{sin1})-\frac{2}{\tau_{if}}x_{fin2}-\frac{1}{{\tau_{if}}^{2}}x_{fin1}$$ (9)

where *x*_*ret*_, *x*_*cc*_, *x*_*tcr*_, *x*_*in*_, *x*_*trn*_, *x*_*py*_, *x*_*ein*_, *x*_*sin*_ and *x*_*fin*_ are the state variables of retinal, cortico-cortical, TCR, IN, TRN, PY, eIN, sIN, fIN neuron populations respectively. *P*_1_(*t*) represents the extrinsic afferent to the thalamic module from the retino-geniculate neuron population in the state of eyes closed and relaxed wakefulness, which is modelled by Gaussian white noise with mean *μ*_*r*_ and variance *φ*_*r*_. *P*_2_(*t*) denotes the cortical module input from neighboring cortical regions, which is also simulated by Gaussian white noise with mean *μ*_*c*_ and variance *φ*_*c*_. The excitatory synaptic strength is expressed by *H*_*e*_. The inhibitory synaptic strength in the thalamic module is represented by *H*_*i*_, and the inhibitory synaptic strength in the cortical module is denoted by *H*_*il*_ or *H*_*if*_. *τ*_*e*_ is the time constant of excitatory postsynaptic potential (PSP). *τ*_*i*_ denotes the time constant of inhibitory PSP in the thalamic module, yet the time constant of inhibitory PSP generated by the sIN or fIN neuronal population is denoted by *τ*_*il*_ or *τ*_*if*_. Sigmoid function S(•) is employed to transmit the membrane potential *V*_*neuron*_: neuron ∈ {*tcr*, *in*, *trn*, *py*, *ein*, *sin*, *fin*} of a postsynaptic neuron population to an average firing rate and is described by the following equation:

$$S(V_{neuron})=\frac{2e_{0}}{1+e^{v(s_{0}-V_{neuron})}}$$ (10)

in which 2*e*_0_ is the maximum discharge rate of neuron populations, *s*_0_ is the firing threshold, *v* refers to the sigmoid steepness parameter. The postsynaptic membrane potential *V*_*neuron*_ is defined as:

$$V_{tcr}=C_{tre}x_{ret1}+C_{tpe}x_{py1}-C_{tii}x_{in1}-C_{tni}x_{trn1}$$ (11)

$$V_{in}=C_{ire}x_{ret1}+C_{ipe}x_{py1}-C_{isi}x_{in1}$$ (12)

$$V_{trn}=C_{nte}x_{tcr1}+C_{npe}x_{py1}-C_{nsi}x_{trn1}$$ (13)

$$V_{py}=C_{pce}x_{cc1}+C_{pte}x_{tcr1}+C_{pxe}x_{ein1}-C_{pli}x_{sin1}-C_{pfi}x_{fin1}$$ (14)

$$V_{ein}=C_{xpe}x_{py1}+C_{xte}x_{tcr1}$$ (15)

$$V_{sin}=C_{lpe}x_{py1}+C_{lte}x_{tcr1}-C_{lfi}x_{fin1}$$ (16)

$$V_{fin}=C_{fpe}x_{py1}+C_{fte}x_{tcr1}-C_{fli}x_{sin1}$$ (17)

in which *C*_*xyz*_ show the synaptic connectivity parameters made by a presynaptic neuron population *y* on a single dendritic terminal of the postsynaptic neuron population *x* with an excitatory or an inhibitory synapse *z*, where *x*, *y* and *z* are defined as follows:

x={t,i,n,p,x,l,f}y={t,i,n,p,x,l,f,r,c,s}z={e,i} (18)

where *t*, *i* and *n* indicate afferent and efferent from TCR, IN and TRN neuron populations, respectively; *p*, *x*, *l* and *f* denote afferent and efferent from PY, eIN, sIN and fIN neuron populations, respectively; the extrinsic inputs from the retinal and cortical regions to the thalamic and the cortical module are denoted by *r* and *c*, respectively; *s* stands for self-input of neuron populations; *e* and *i* represent excitatory and inhibitory synapses, respectively.

Note that the differential equations of the modified TCT model are simulated by the Euler technique in Matlab 2016b. Unless specially stated, the synaptic connectivity parameters and the other parameters in this model are given in Tables 1 and 2, respectively. In this work, we are interested in the output of the thalamic module, which is presented by the membrane potential of the TCR neuron population. For each set of given parameters, the model output is an average of 50 independent realizations to guarantee statistical accuracy. In order to obtain the peak power density within the alpha band (7.5–13.5Hz) of the thalamic module output, the power spectral analysis is carried out as follows: 1) The thalamic module output is bandpass filtered by employing a butterworth filter of order 10 with lower and upper cut-off frequencies of 1 and 50 Hz, respectively; 2) The power spectral density is obtained using a Welch periodogram method with a hamming window; 3) The peak of the power spectral density within the alpha band is extracted.

**Table 1:** Parameters of synaptic connection between different neuron populations in the model.

Module	Objective (to)	Origin (from)	Connectivity parameter	Value
Thalamic module	TCR	Retinal	*C*_*tre*_	7.1
		IN	*C*_*tii*_	15.45
		TRN	*C*_*tni*_	15.45
		PY	*C*_*tpe*_	62
	IN	Retinal	*C*_*ire*_	47.4
		IN	*C*_*isi*_	23.6
		PY	*C*_*ipe*_	29
	TRN	TCR	*C*_*nte*_	35
		TRN	*C*_*nsi*_	15
		PY	*C*_*npe*_	50
Cortical module	PY	Cortical	*C*_*pce*_	1
		TCR	*C*_*pte*_	80
		eIN	*C*_*pxe*_	108
		sIN	*C*_*pli*_	33.75
		fIN	*C*_*pfi*_	108
	eIN	TCR	*C*_*xte*_	100
		PY	*C*_*xpe*_	135
	sIN	TCR	*C*_*lte*_	40
		PY	*C*_*lpe*_	33.75
		fIN	*C*_*lfi*_	13.5
	fIN	TCR	*C*_*fte*_	40
		PY	*C*_*fpe*_	40.5
		sIN	*C*_*fli*_	13.5

**Table 2:** Values of other parameters in this model that sourced from works [41, 43].

Parameter	Unit	Module	Value
*v*	mV^-1^	Thalamic/Cortical	0.56
*e*_0_	s^-1^	Thalamic/Cortical	2.5
*s*_0_	MV	Thalamic/Cortical	6
*μ*_*r*_	Spikes per second(sps)	Thalamic	5
*φ*_*r*_	sps^2^	Thalamic	0.05
*μ*_*c*_	sps	Cortical	13
*φ*_*c*_	sps^2^	Cortical	0.05
*H*_*e*_	mV	Thalamic	3.25
		Cortical	2.7
*H*_*i*_	mV	Thalamic	22
*H*_*il*_	mV	Cortical	4.5
*H*_*if*_	mV	Cortical	39
*τ*_*e*_	ms	Thalamic	10
		Cortical	25
*τ*_*i*_	ms	Thalamic	25
*τ*_*il*_	ms	Cortical	50
*τ*_*if*_	ms	Cortical	3
T	Normalized	Thalamic	100

The values of connectivity parameters in the thalamic module are selected according to the physiological data and express as a percentage of total synapses T convergent on the terminal of the thalamic neuron populations [42]. The values of synaptic strengths in the cortical module are obtained from the work [41]. The values of synaptic connections between the thalamic and the cortical module are chosen based on the Ref. [43].

## Main results

Neuroanatomical studies have found that in some neuronal system diseases including Alzheimer’s disease, the synapses of neurons in the pathopoiesis brain area will change [24]. One typical case is that the number of synapses will decrease, i.e., synapse loss. Loss of synapses leads to a decrease in the strength of synaptic connections. It is reasonable to infer that there is a decrease in synapse connections between different neuron populations in our TCT model in the neuropathological condition of AD. Thus, in the following firstly we vary the synaptic connectivity parameters of the model to simulate the effects of AD on aberrations of synaptic connectivity and explore the correlation between neuronal synaptic behavior and abnormal alpha rhythm by means of power spectral analysis. In particular, we decrease some typical synaptic connectivity parameters to mimic the hallmark neuropathological condition of synapse loss in AD, where the considered synapse parameters include not only the ones in the original TCT model but also those related with two newly incorporated biologically plausible ingredients of disinhibition property between different inhibitory interneurons as well as the full relay function of thalamic relay nucleus. In detail, they are the TCR to fIN excitatory connectivity (*C*_*fte*_), the fIN to sIN inhibitory connectivity (*C*_*lfi*_), the eIN to PY excitatory connectivity (*C*_*pxe*_) and the IN to TCR inhibitory connectivity (*C*_*tii*_). Then the dynamical mechanism underlying the alpha rhythmic changes is analyzed using nonlinear dynamical behavioral method.

### Changing the excitatory connectivity parameter from TCR to fIN

In this part, the influence of the excitatory synaptic connection from the TCR neuron population of the thalamic module to the fIN neuron population of the cortical module on the power spectra over the alpha band is first investigated. Fig 2A illustrates how the peak power density within the alpha band changes as the excitatory connectivity parameter from TCR to fIN (*C*_*fte*_) is varied in the range of 25–45. From this figure, one can observe that upon decreasing *C*_*fte*_, the plot of peak power density is fairly flat until *C*_*fte*_ arrives at a certain value of about 35, then it falls sharply until *C*_*fte*_ ≈ 31.5, after which the peak power density does not decrease anymore and basically tends to be stable. To visualize this result, the corresponding power spectral density curves for some typical synaptic strengths (for example *C*_*fte*_ = 30, 32, 34, 36) are also illustrated in Fig 2B. Obviously, within the alpha band the smaller the excitatory connectivity parameter *C*_*fte*_, the lower the peak of power density, which is consistent with the observation of a decrease of the peak power density in the alpha band shown in Fig 2A. The decrease of the peak power density within the alpha band implies that there is a slowing of the alpha band upon decreasing the excitatory synaptic connectivity *C*_*fte*_. Note that we decrease the synaptic connectivity parameter to mimic the hallmark neuropathological condition of synapse loss in AD, thus the above phenomenon of alpha rhythm slowing is consistent with the electrophysiological experimental results of EEG characteristics in AD patients [8, 10].

**Fig 3:**
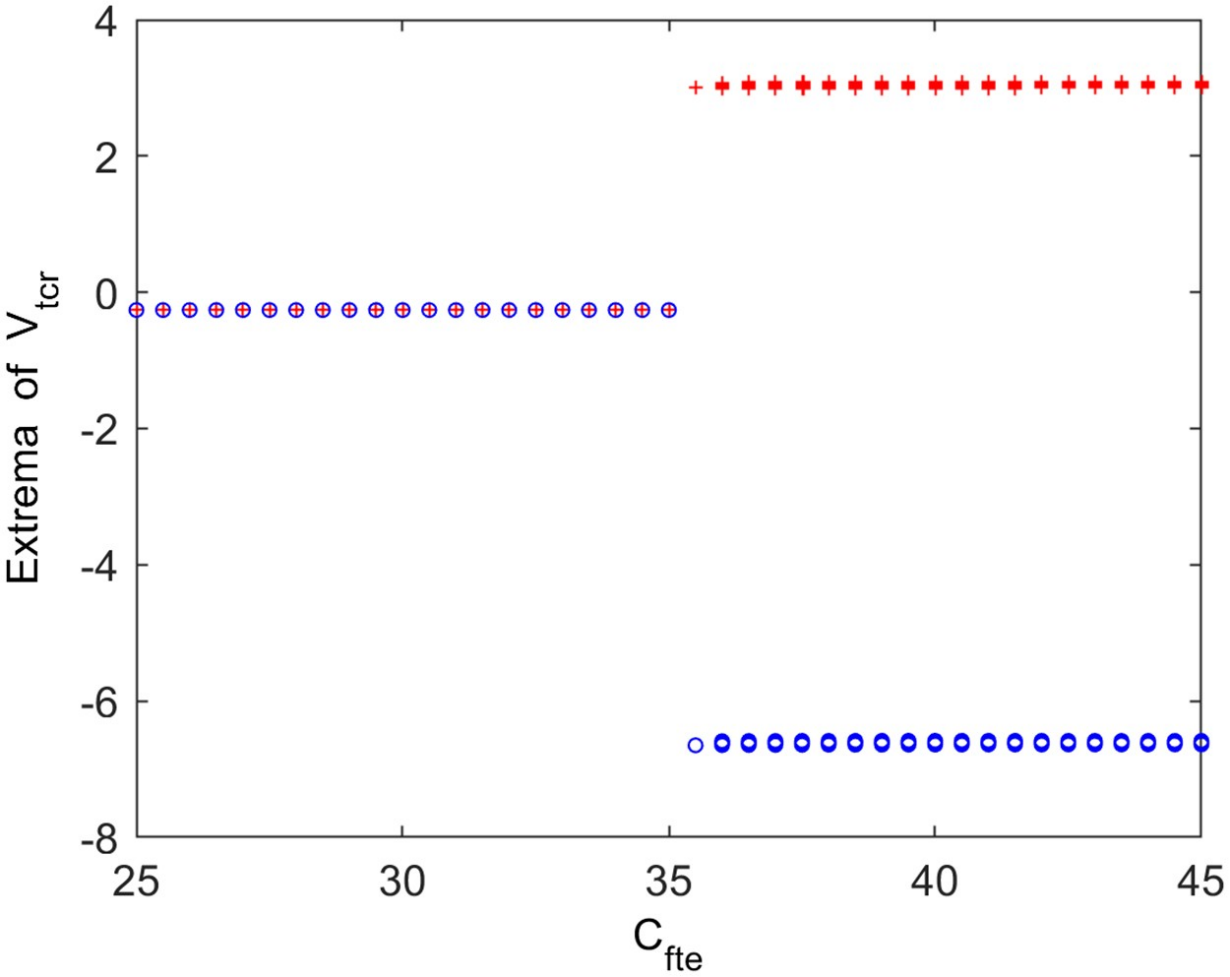
Bifurcation diagram of the thalamic module output’s extrema for varying values of connectivity parameter *C*_*fte*_. Red and blue points represent the local maximum and minimum of the thalamic module output, respectively.

**Fig 4:**
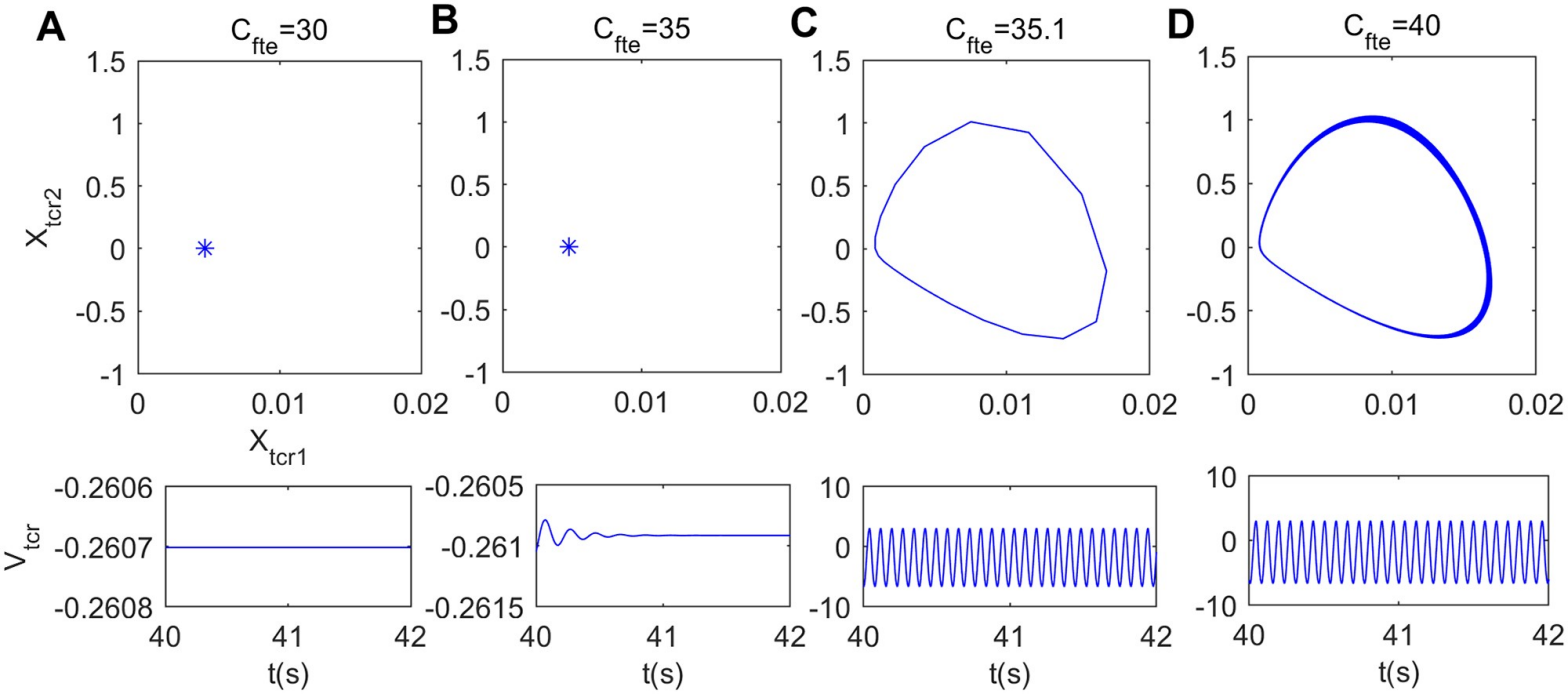
Phase diagrams (top panels) and the corresponding time series diagrams (bottom panels) of the thalamic module. (A) *C*_*fte*_ = 30, (B) *C*_*fte*_ = 35, (C) *C*_*fte*_ = 35.1, (D) *C*_*fte*_ = 40.

Furthermore, the technique of nonlinear dynamics including bifurcation diagram and phase analysis is employed to explore the underlying dynamical mechanism behind the above alpha rhythmic changes induced by the excitatory connectivity parameter *C*_*fte*_ from TCR to fIN. Fig 3 displays the bifurcation diagram of the extrema of the thalamic module output in the process of increasing *C*_*fte*_. It can be seen that there is only one extreme in the bifurcation plot when *C*_*fte*_ is not more than 35, meaning that V_*tcr*_ stabilizes to an equilibrium point and the thalamic module is in a point attractor mode. While two extrema of maximum and minimum emerge in the bifurcation diagram when *C*_*fte*_ is greater than 35, suggesting that the thalamic module undergoes a Hopf bifurcation and turns into a limit cycle mode. To verify the above bifurcation phenomenon, Fig 4 show the phase plots and the corresponding time series curves when *C*_*fte*_ takes some typical values. In detail, when *C*_*fte*_ is below the bifurcation point (for example *C*_*fte*_ = 30, 35), there is a point attractor in phase space and the output of thalamic module V_*tcr*_ settles to a constant after a short period of transition. Once *C*_*fte*_ is beyond the bifurcation point (see for example *C*_*fte*_ = 35.1, 40), however, there is an isolated closed curve on phase space and the thalamic model performs oscillatory motion fluctuating between its maximum and minimum. Obviously, with decreasing the excitatory connectivity parameter *C*_*fte*_ from TCR to fIN, the dynamics of the thalamic module shifts from a limit cycle mode to a point attractor mode. This dynamics change may be related with the decreased trend of the peak power density, i.e., the slowing of the alpha rhythm in the modified TCT model upon decreasing the excitatory connectivity parameter *C*_*fte*_ from TCR to fIN.

**Fig 5:**
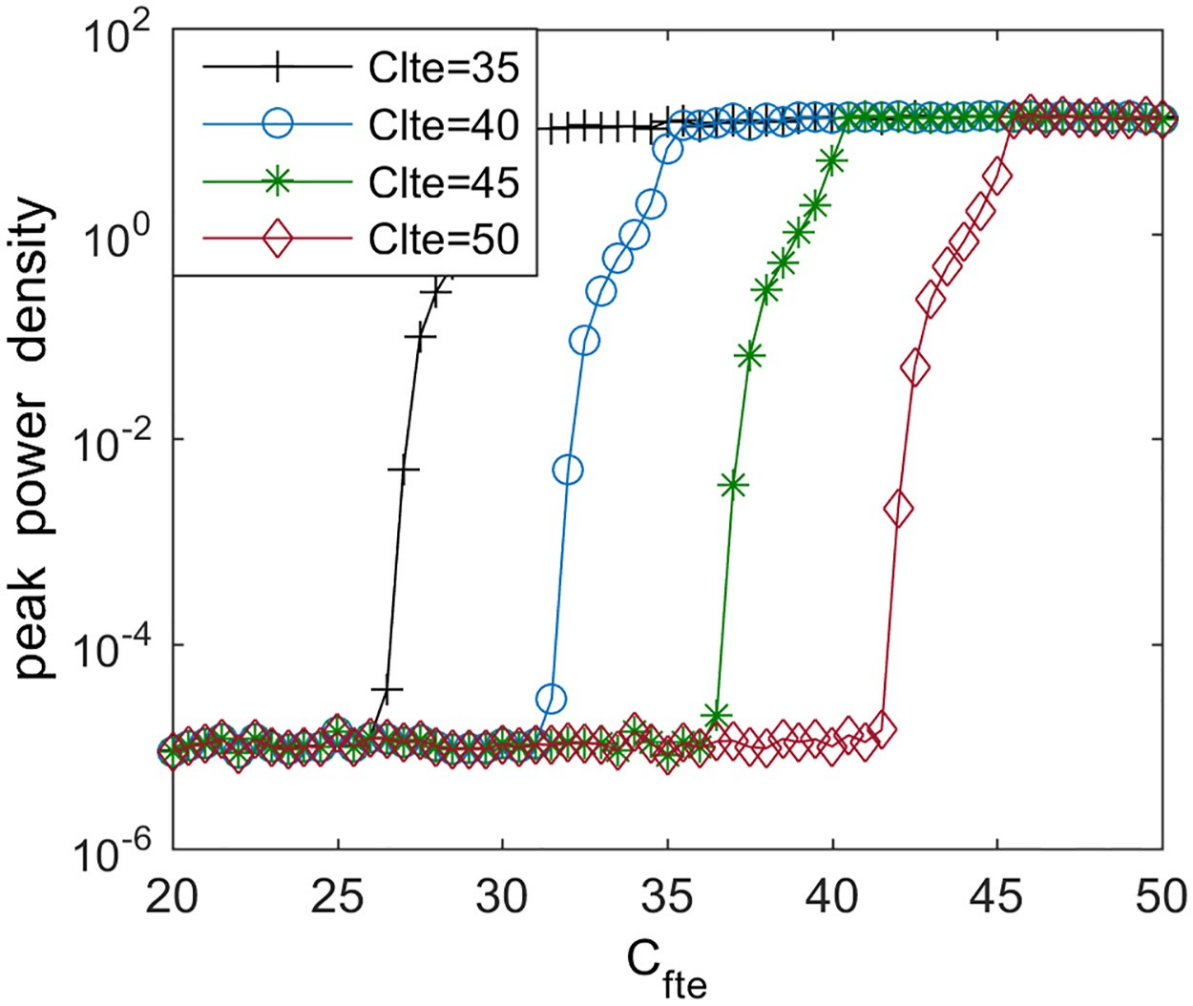
Dependence of the peak power density within alpha band on synaptic connectivity *C*_*fte*_ for different parameters of *C*_*lte*_. Here, *C*_*lte*_ = 35, 40, 45, 50.

Note that the excitatory connectivity parameter from TCR to sIN is fixed at *C*_*lte*_ = 40 in the above discussion. We wonder whether the slowing of the alpha rhythm induced by decreasing the excitatory connectivity parameter *C*_*fte*_ can still emerge when this parameter *C*_*lte*_ is changed. For this purpose, the dependence of the peak power density within the alpha band on *C*_*fte*_ for various values of *C*_*lte*_ are illustrated in Fig 5. Clearly, the curves of peak power density have similar shapes for different *C*_*lte*_, which implies that the slowing of the alpha rhythm induced by connectivity parameter *C*_*fte*_ is robust to synaptic connection *C*_*lte*_. On the same time, upon increasing *C*_*lte*_ a right shift of the decreased trend is notable, which demonstrates that the slowing of the alpha rhythmic activity during the decrease of the excitatory connectivity parameter *C*_*fte*_ is preferred to occur with the increase of the excitatory connectivity parameter *C*_*lte*_ from TCR to sIN.

### Changing the inhibitory connectivity parameter from fIN to sIN

**Fig 6:**
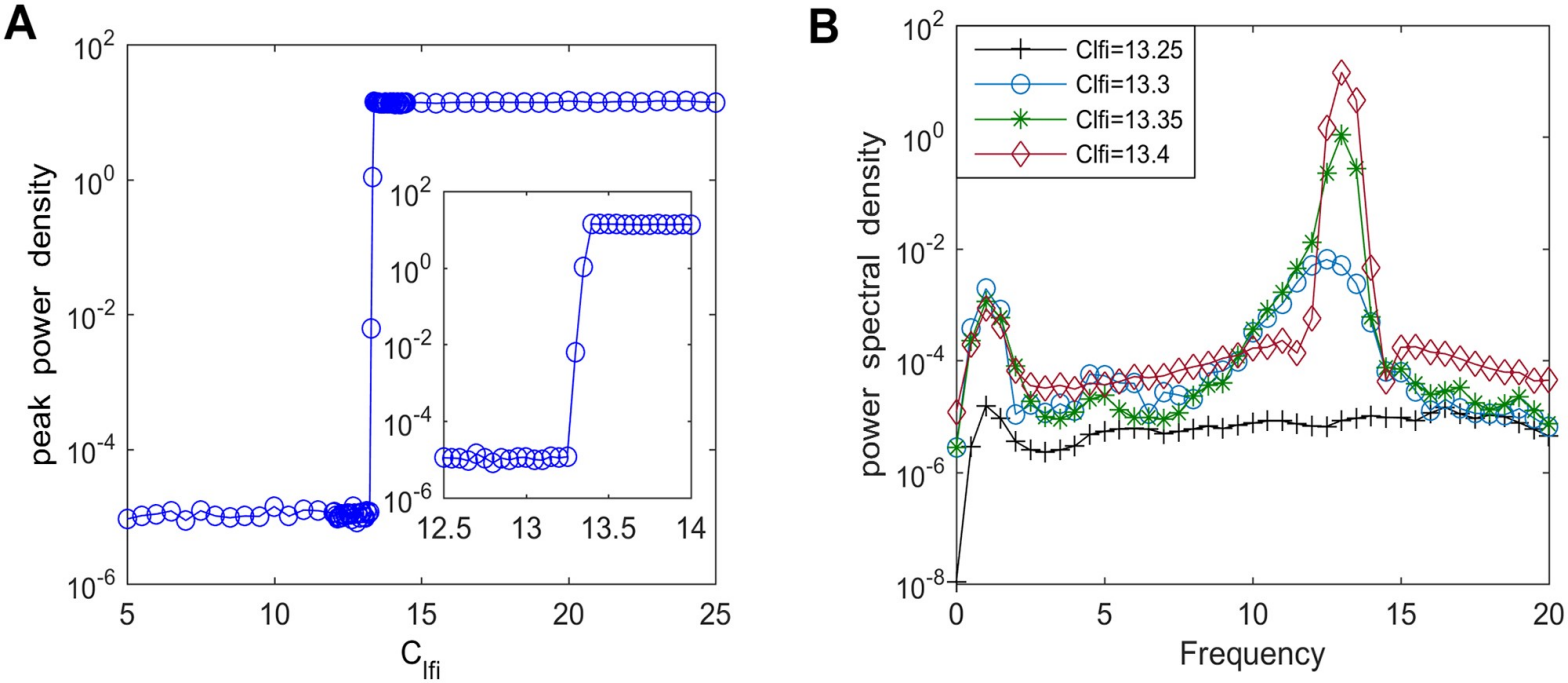
The peak power density and the corresponding power spectral density for varying values of *C*_*lfi*_. (A) The peak power density within the alpha band of the thalamic module. (B) The corresponding power spectral density for different values of *C*_*lfi*_ such as *C*_*lfi*_ = 13.25, 13.3, 13.35, 13.4.

The previous works have indicated that the inhibitory synaptic connectivity plays a crucial role in the slowing of the alpha frequency band in the thalamocortical circuitry [17, 44]. Inspired by this fact, how the inhibitory pathway connectivity from the fIN neuron population of the cortical module to the sIN neuron population of the cortical module affects the power spectral content within the alpha band in the modified TCT model is discussed. Fig 6A delineates the relationship between the peak power density over the alpha band and the inhibitory connectivity parameter from fIN to sIN (*C*_*lfi*_) when *C*_*lfi*_ is varied in the range of 5–25. The peak power density fluctuates slightly as *C*_*lfi*_ is initially decreased. Interestingly, its downward trend is quite significant when *C*_*lfi*_ changes from about 13.5 to 13.1. Afterwards, the peak power density no longer decreases anymore and tends to be stabilized upon further decreasing *C*_*lfi*_. To illustrate this result more detail, the corresponding power spectral density curves for some different values of *C*_*lfi*_ (such as *C*_*lfi*_ = 13.25, 13.3, 13.35, 13.4) are described in Fig 6B. From this figure, on can find that the greater the *C*_*lfi*_, the higher the peak of the power spectral density within the alpha band, which confirms the result obtained in Fig 6A. Thus, it can be concluded that the peak power density over the alpha band tends to decline by decreasing inhibitory synaptic connection *C*_*lfi*_, which suggests the slowing of alpha band emerges when mimicking the hallmark neuropathological condition of synapse loss in AD, i.e., a prominent feature of EEG in AD patients observed in electrophysiological experiments [8, 10].

**Fig 7:**
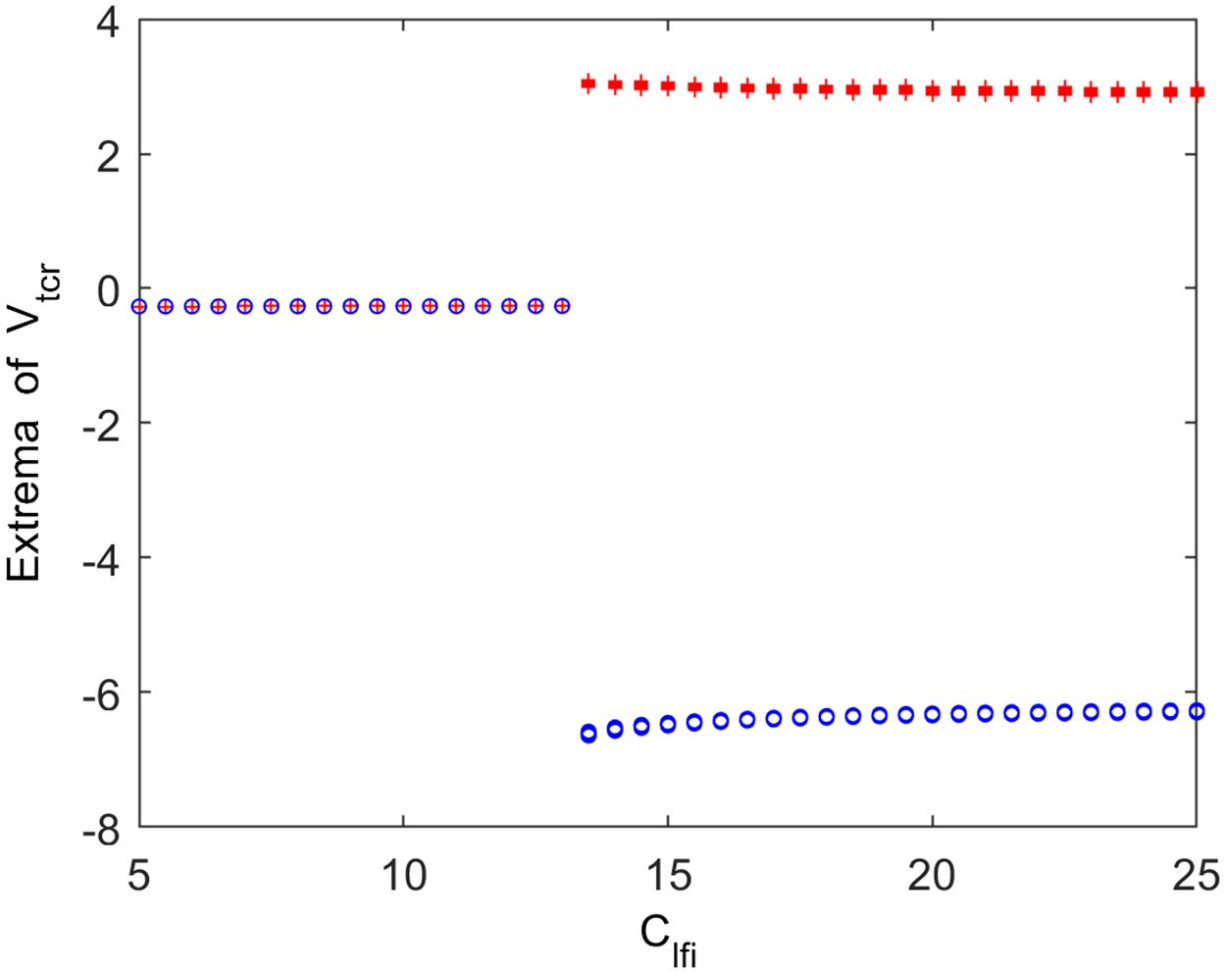
Bifurcation analysis of the thalamic module’s extrema for varying synaptic connection *C*_*lfi*_. Red and blue points indicate the local maximum and minimum of the thalamic output, respectively.

**Fig 8:**
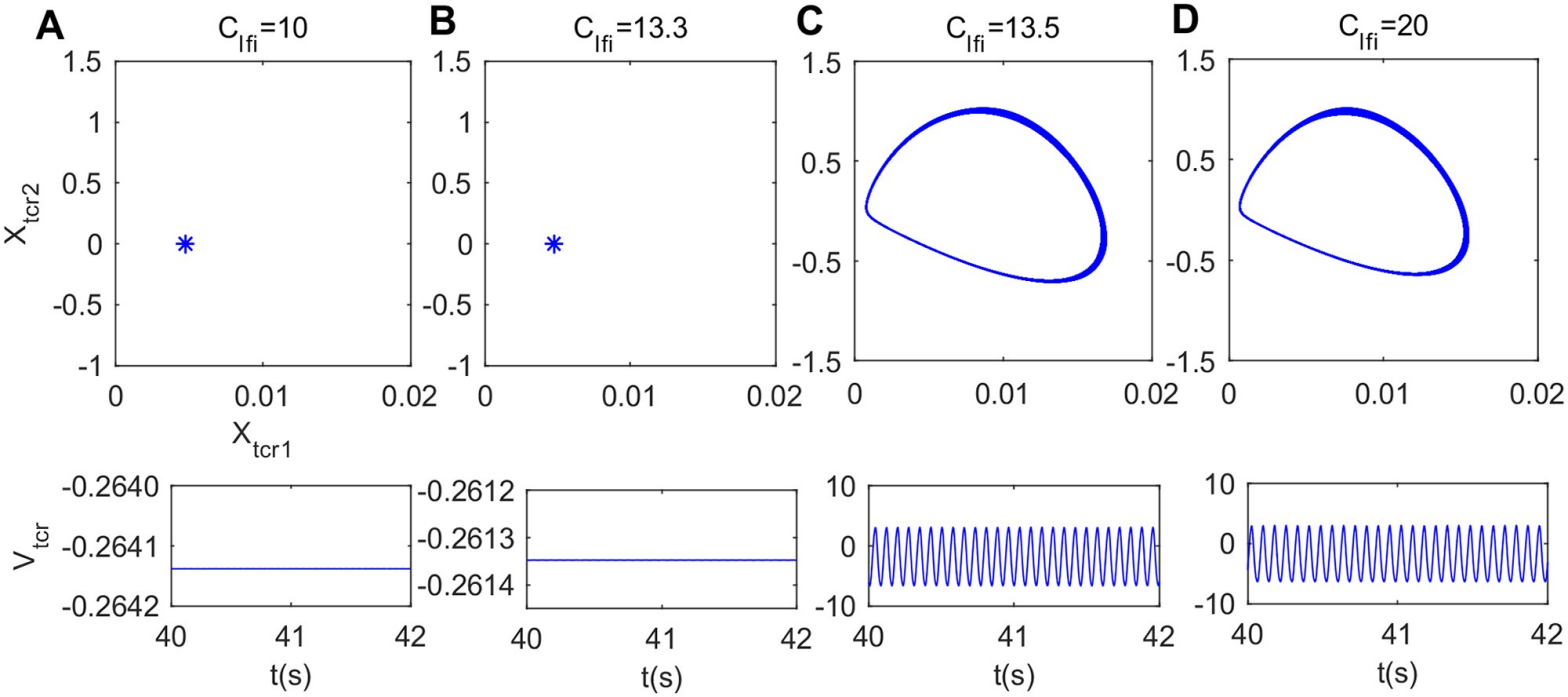
Phase plots (top panels) and the corresponding time series plots (bottom panels) of the thalamic module when *C*_*lfi*_ takes different values. (A) *C*_*lfi*_ = 10, (B) *C*_*lfi*_ = 13.3, (C) *C*_*lfi*_ = 13.5, (D) *C*_*lfi*_ = 20.

It is interesting that the peak power density within the alpha band begins to decrease when the inhibitory synaptic connection from fIN to sIN (*C*_*lfi*_) approaches to a critical value (*C*_*lfi*_ ≈ 13.5). We cannot help wonder that what is the underlying dynamical mechanism of this change in the power spectral content over the alpha frequency band. To solve this problem, the bifurcation analysis and phase analysis are employed. Fig 7 illustrates the bifurcation behavior of the thalamic module’s extrema with increasing connectivity parameter *C*_*lfi*_. Through careful observation, it is clear that only one extreme emerges in the bifurcation diagram for *C*_*lfi*_ < 13.5, revealing that the thalamic module is in a point attractor mode. However, for *C*_*lfi*_ ≥ 13.5, a maximum together with a minimum appears in the bifurcation diagram, which indicates that the thalamic module undergoes a Hopf bifurcation and then transfers to a limit cycle mode. In order to vividly depict the thalamic module dynamical behavior, the phase orbits and the corresponding time series plots are displayed in Fig 8. From panel (A) to panel (D), *C*_*lfi*_ is in turn 10, 13.3, 13.5, 20. When *C*_*lte*_ < 13.5, the module tends to a attract point on phase plane and *V*_*tcr*_ gradually converges to a constant (please see panel (A) and (B)). However, for values of *C*_*lfi*_ ≥ 13.5, there is an isolated loop in phase plane and the corresponding time series plot presents the periodic motion of the thalamic module (please see panel (C) and (D)). The above results mean that with the decrease of inhibitory parameter *C*_*lfi*_ from fIN to sIN, the thalamic module throws out of a limit cycle mode and enters into a stable point attractor mode, which may induce the reduction of the peak power density over the alpha band, i.e., a slowing of the alpha rhythm in the modified TCT model with the decrease of inhibitory synaptic activity *C*_*lfi*_.

### Changing the excitatory connectivity parameter from eIN to PY

**Fig 9:**
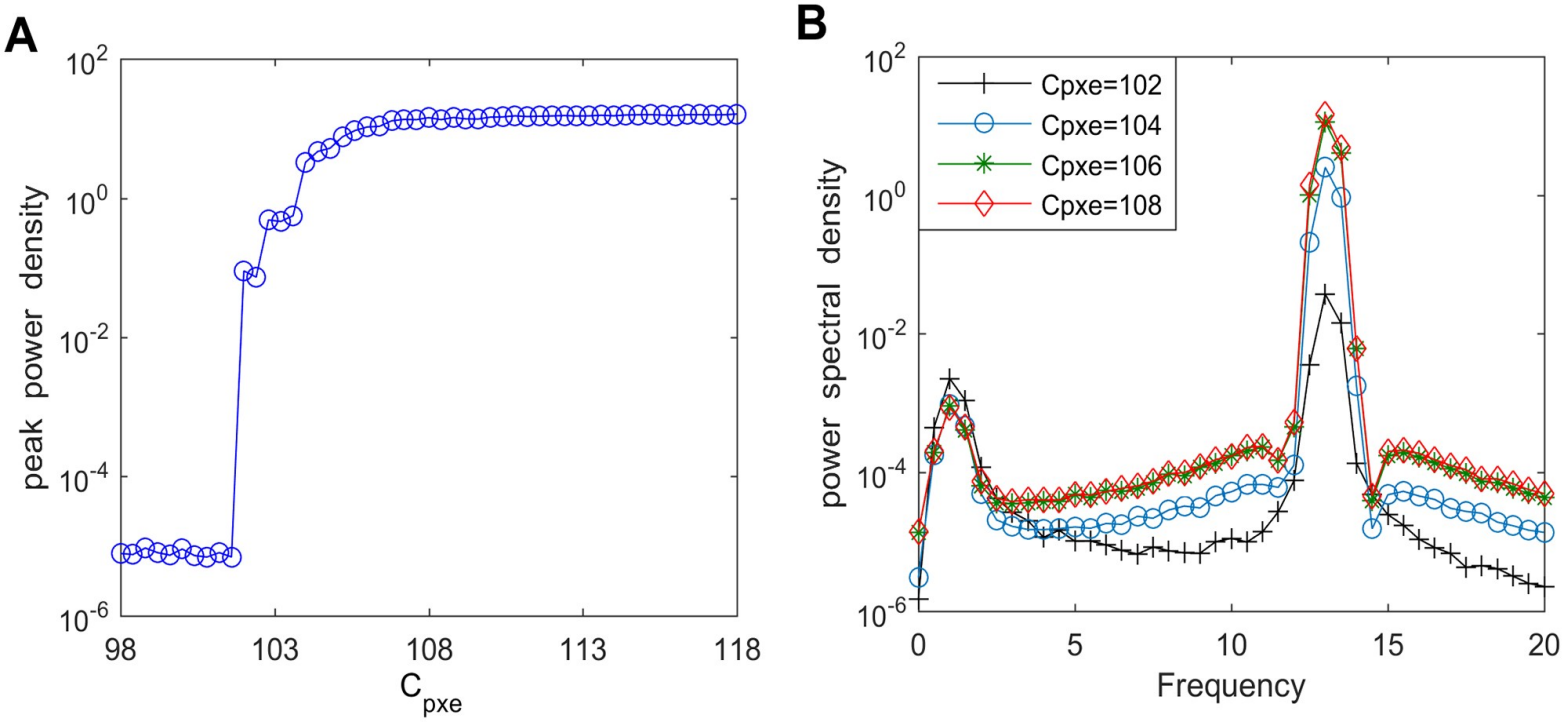
The peak power density and the corresponding power spectral density for varying values of *C*_*pxe*_. (A) The peak power density within the alpha band of the thalamic output. (B) The corresponding power spectral density for different connectivity parameters such as *C*_*pxe*_ = 102, 104, 106, 108.

The above discussion reveals the influence of the newly introduced synaptic connectivity parameters on the alpha band power in this modified TCT model. In the following, we analyze how the synaptic parameters existing in the original TCT model affect the power density within the alpha band. Firstly, the effect of excitatory synaptic parameter from the eIN population of the cortical module to PY neuron population of the cortical module on the alpha rhythmic activity is investigated. When the connectivity parameter *C*_*pxe*_ from eIN to PY is varied from 98 to 118, the peak power density with alpha band is depicted in Fig 9A. It is clear that the peak power density almost remains unchanged with the first decrease of *C*_*pxe*_, then it decreases rapidly when *C*_*pxe*_ decreases from about 108 to 101.6, after that the peak power density ceases to decrease anymore. Subsequently, the corresponding power spectral density curves for the case of *C*_*pxe*_ = 102, 104, 106, 108 are employed to validate the above result. From Fig 9B one can see that the decreased *C*_*pxe*_ leads to a decrease in the power spectral density within the alpha band, which accords with the reduction of the peak power density within the alpha band presented in Fig 9A. The above phenomenon implies that decreasing synaptic activity *C*_*pxe*_ to mimic the hallmark neuropathological condition of synapse loss in AD can lead to a slowing of the alpha rhythmic content, which is consistent with the electrophysiological experimental results on EEG characteristics in AD patients [8, 10].

**Fig 10:**
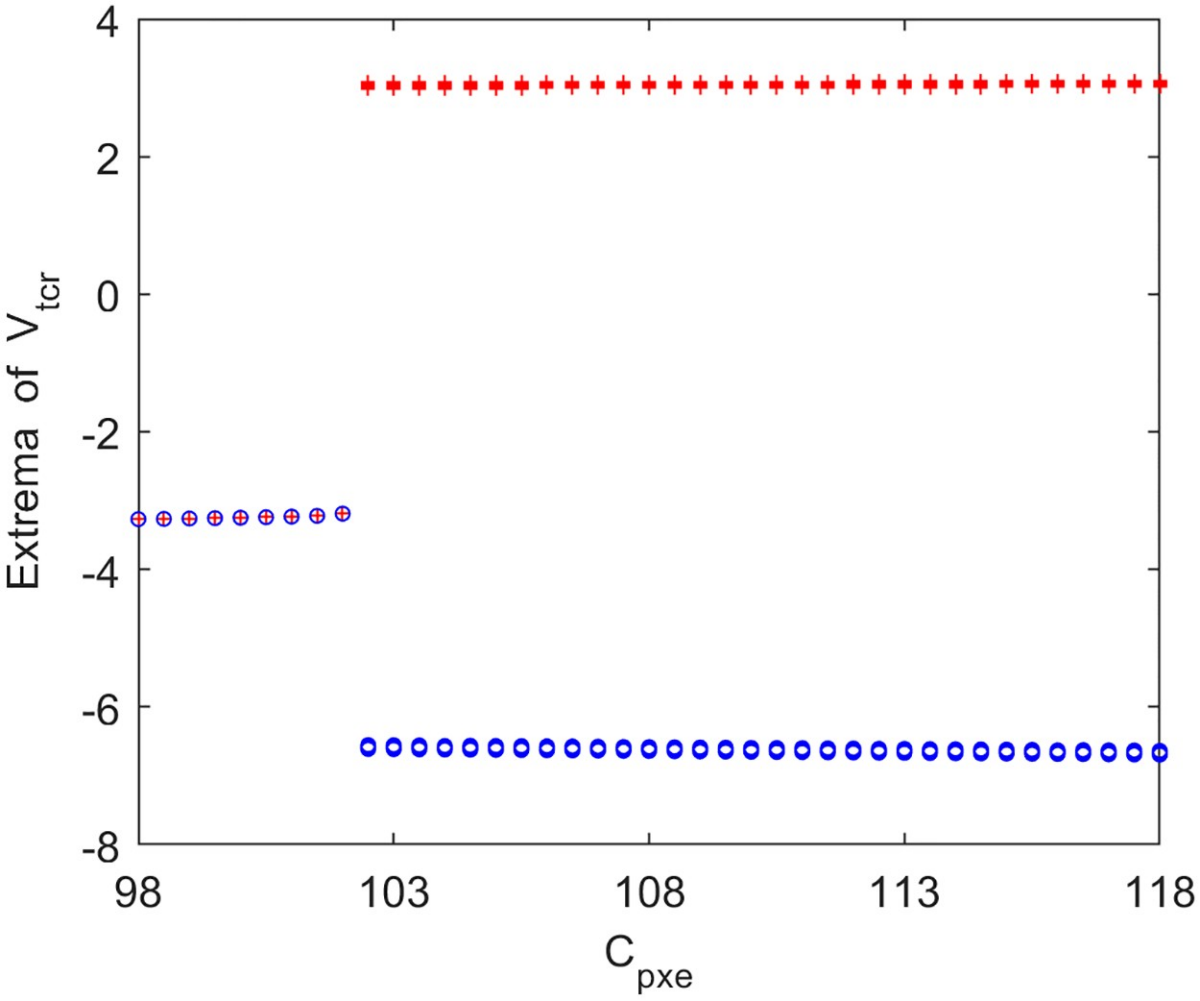
Bifurcation analysis of the thalamic module’s extrema for varying synaptic connection *C*_*pxe*_. Red and blue points indicate the local maximum and minimum of the thalamic output, respectively.

**Fig 11:**
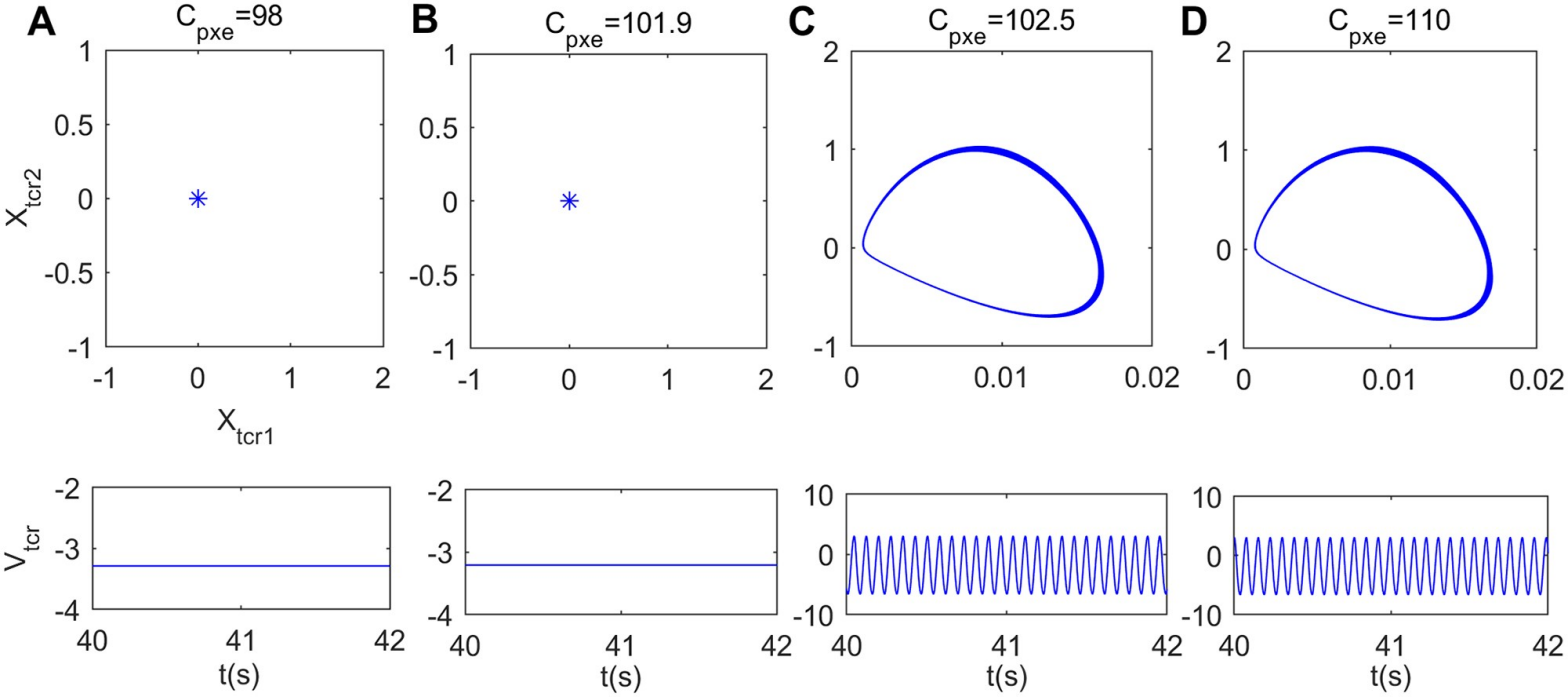
Phase plots (top panels) and the corresponding time series plots (bottom panels) of the thalamic module when *C*_*pxe*_ takes different values. (A) *C*_*pxe*_ = 98, (B) *C*_*pxe*_ = 101.9, (C) *C*_*pxe*_ = 102.5, (D) *C*_*pxe*_ = 110.

In what follows, the underlying dynamical mechanism for the reduction of power spectral content within the alpha band induced by excitatory pathway connectivity from the eIN to PY (*C*_*pxe*_) is explored by nonlinear dynamical behavioral method. The bifurcation plot of the thalamic output’s extrema with increasing *C*_*pxe*_ is shown in Fig 10. One can see that extrema in the bifurcation diagram change from one to two when *C*_*pxe*_ ≈ 102.5, implying that the thalamic module undergoes a Hopf bifurcation and transits from a point attractor mode to a limit cycle model. To illustrate the above bifurcation phenomenon, Fig 11 describe the phase orbits and the corresponding time series plots when the connection parameter *C*_*pxe*_ takes some typical values such as *C*_*pxe*_ = 96, 101.9, 102.5, 110. From these figures, it can be found that when *C*_*pxe*_ increases to an appropriate value (*C*_*pxe*_ ≈ 102.5), the phase orbit of the model changes from a point attractor to a closed curve, and the thalamic module’s dynamics transforms from an equilibrium point to oscillatory motion. Clearly, upon decreasing the excitatory connectivity parameter *C*_*pxe*_ from eIN to PY, the dynamics of the thalamic module transfers from a limit cycle mode to a point attractor mode, which may be related with the reduction of the peak power density within the alpha band, i.e., the modified TCT model shows a slowing of the alpha rhythm with decreasing the excitatory synaptic activity *C*_*pxe*_.

### Changing the inhibitory connectivity parameter from IN to TCR

**Fig 12:**
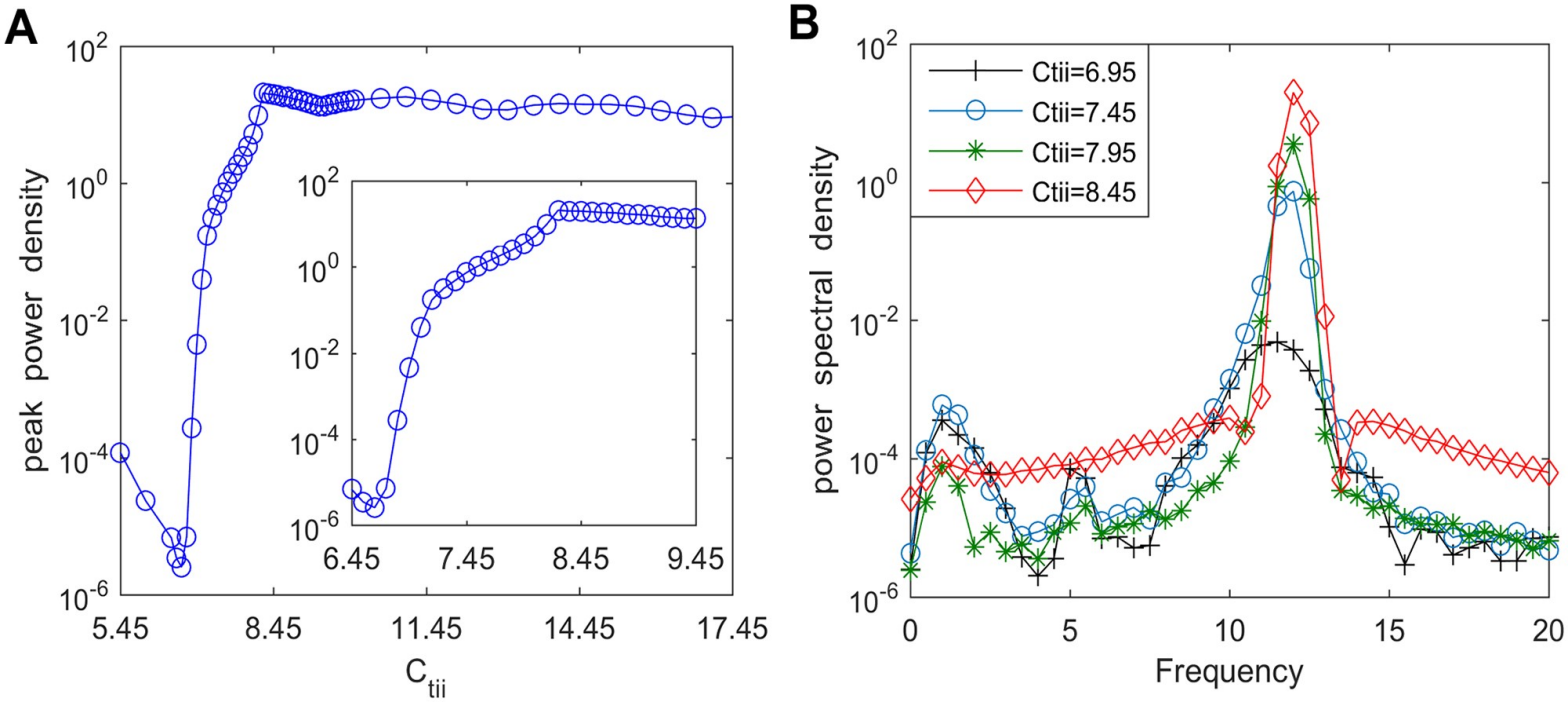
The peak power density and the corresponding power spectral density for varying values of *C*_*tii*_. (A) The peak power density within the alpha band of the thalamic output. (B) The corresponding power spectral density for different connectivity parameters such as *C*_*tii*_ = 6.95, 7.45, 7.95, 8.45.

In this part, we first carry out the power spectral analysis by varying the inhibitory synaptic activity *C*_*tii*_ from the IN population to the TCR neuron population within the thalamic module. The dependence of peak power density with the alpha band on connectivity parameter *C*_*tii*_ over the range of 5.45–17.45 is illustrated in Fig 12A. Obviously, the peak power density decreases quickly when *C*_*tii*_ decreases from about 8.25 to 6.45. Subsequently, to validate the above result, the power spectral density curves for different values of *C*_*tii*_ = 6.95, 7.45, 7.95, 8.45 are described in Fig 12B. It is clear that a smaller value of *C*_*tii*_ leads to a lower peak power density with alpha band, which verifies the above phenomenon described in Fig 12A. Thus, it can be concluded that the peak power density presents a descending trend with the decrease of synaptic connection *C*_*tii*_. Then one can believe that a decrease in synaptic connectivity *C*_*tii*_ related with synapse loss in AD can induce the slowing of alpha rhythmic content, which agrees with the EEG hallmark of AD patients obtained from the electrophysiological experimental results [8, 10].

**Fig 13:**
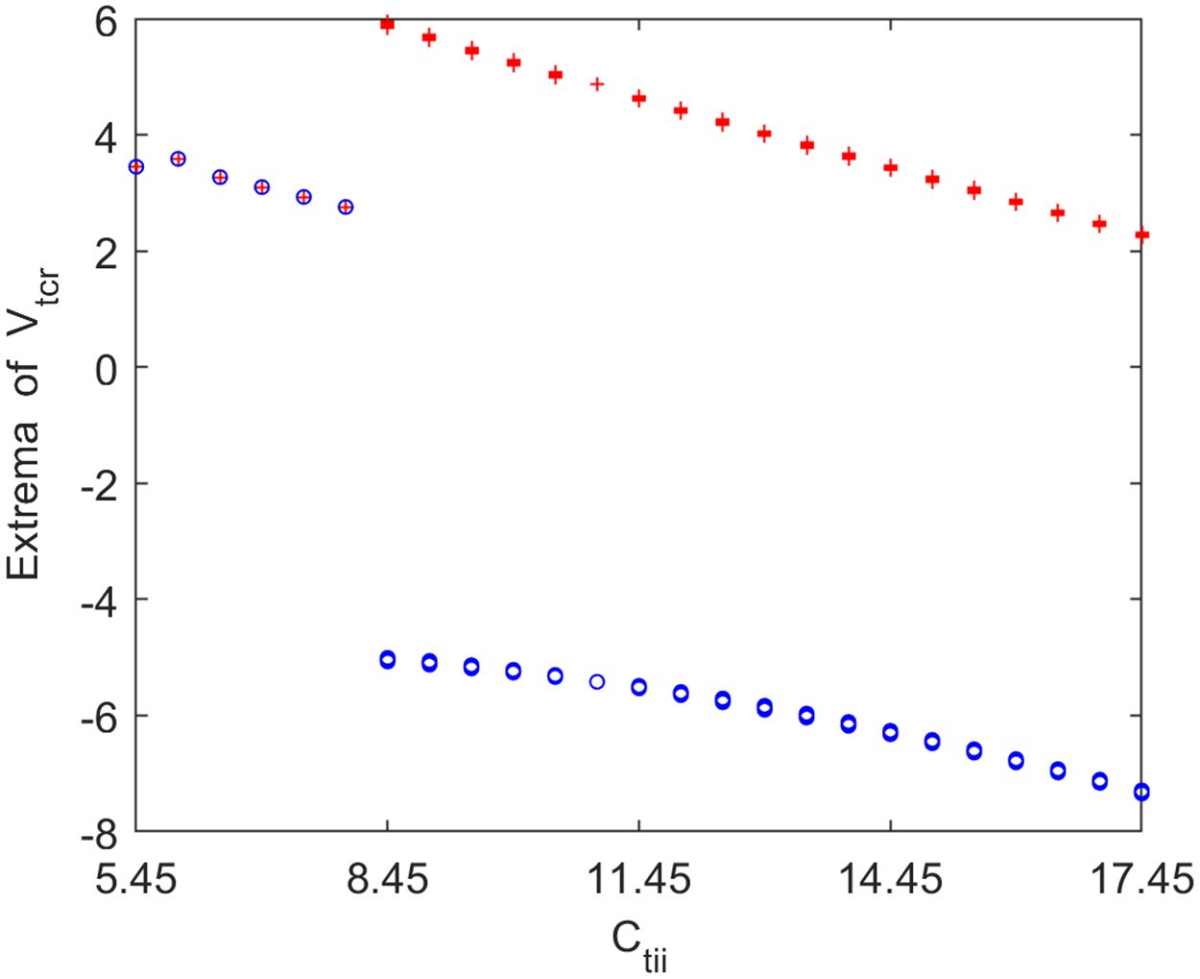
Bifurcation analysis of the thalamic module’s extrema for varying synaptic connection *C*_*tii*_. Red and blue points indicate the local maximum and minimum of the thalamic output, respectively.

**Fig 14:**
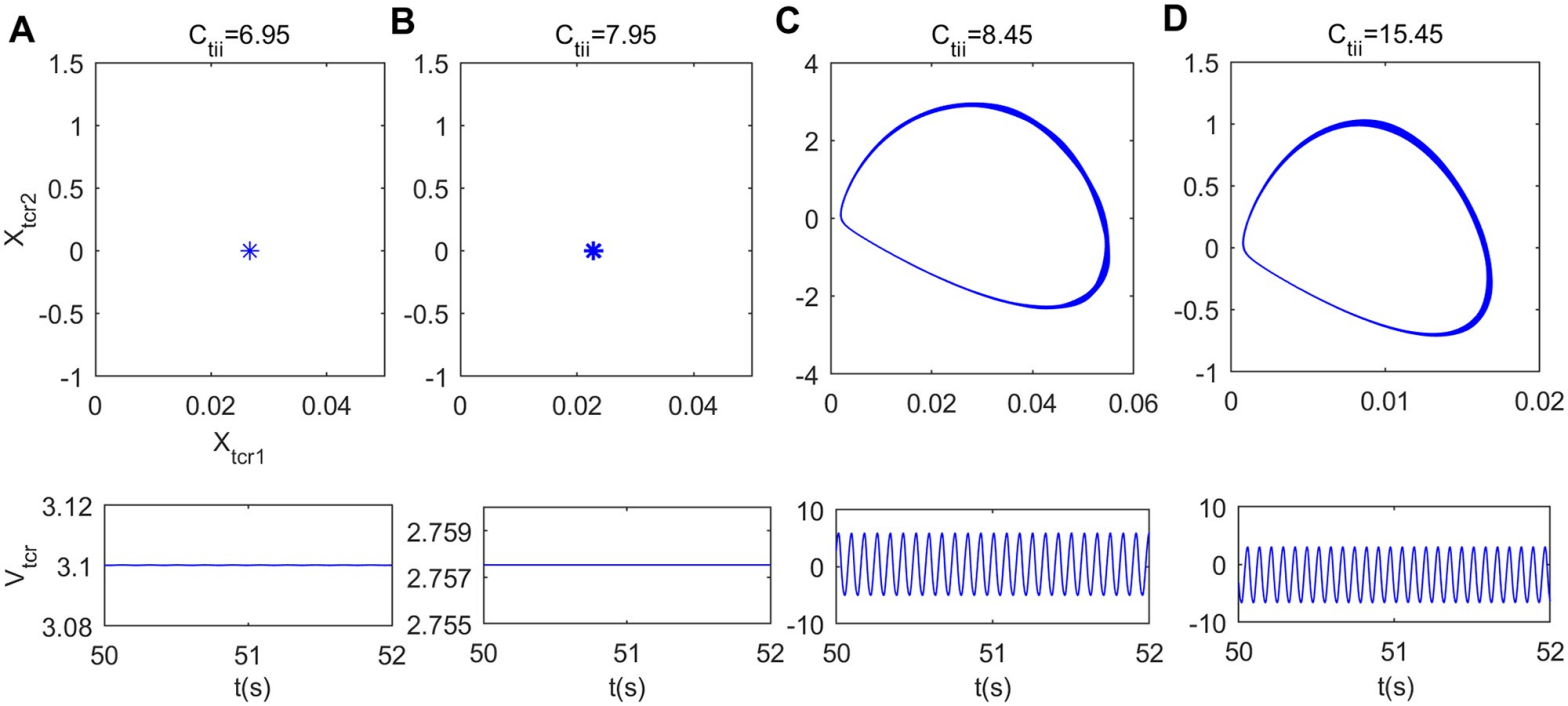
Phase plots (top panels) and the corresponding time series plots (bottom panels) of the thalamic module when *C*_*tii*_ takes different values. (A) *C*_*tii*_ = 6.95, (B) *C*_*tii*_ = 7.95, (C) *C*_*tii*_ = 8.45, (D) *C*_*tii*_ = 5.45.

Furthermore, the underlying dynamical mechanism of alpha rhythm slowing induced by inhibitory connectivity from IN to TCR (*C*_*tii*_) is explored by means of nonlinear dynamical analysis. Fig 13 exhibits the bifurcation behavior of the thalamic module’s extrema upon increasing synaptic connection *C*_*tii*_. From this figure, one can find that when *C*_*tii*_ reaches a specific value (*C*_*tii*_ ≈ 8.45), the dynamical behavior of the thalamic module undergoes a Hopf bifurcation and shifts from the point attractor mode to the limit cycle mode. In order to more vividly characterize the bifurcation process of the module, Fig 14 display the phase orbits and the corresponding time series curves of the thalamic module when *C*_*tii*_ takes some values such as *C*_*tii*_ = 6.95, 7.95, 8.45, 15.45. It can be found that the dynamics of the thalamic module changes from a stable point to oscillatory motion. The above results illustrate that the dynamics of the thalamic module changes from a limit cycle mode to a point attractor mode with a decrease in connectivity parameter *C*_*tii*_, which may be related with the reduction in the peak power density, i.e., the slowing of the alpha rhythmic content in the modified TCT model with the decrease of inhibitory synaptic connection *C*_*tii*_.

## Conclusion and discussion

In view of the two key ingredients including the mutual effects between different inhibitory interneurons and the full relay function of the thalamic relay nucleus, this work firstly constructs a more biologically plausible TCT computational model of thalamo-cortico-thalamic neuronal circuitry. By decreasing synaptic connectivity parameters to mimic the hallmark neuropathological condition of synapse loss in AD, then we explore the correlation between neuronal synaptic behavior and abnormal alpha rhythm by means of power spectral analysis. Numerical results reveal the remarkable role of excitatory synaptic pathways from TCR to fIN (*C*_*fte*_) ae well as from eIN to PY (*C*_*pxe*_) in making the abnormality of power spectrum in this modified TCT model. In detail, upon decreasing excitatory connectivity parameter *C*_*fte*_ and *C*_*pxe*_, the thalamic module shows alpha frequency band slowing represented by a decrease in the peak power density over the alpha band. On the same time, the decreased inhibitory connectivity parameters from fIN to sIN (*C*_*lfi*_) and from IN to TCR (*C*_*tii*_) can also induce the slowing of the alpha band. These interesting results obtained in this modified TCT model highlight the phenomenon of excitatory synaptic connectivity induced slowing of alpha rhythmic activity, which enriches the existing research on changes of alpha rhythm associated with synaptic connection activity in the alpha rhythm model and the modified versions in the literatures [17, 37, 44]. What’s more, the results indicate that the dependence of peak power density within the alpha band on the synaptic connectivity parameter *C*_*fte*_ are robust to the variation of synaptic activity *C*_*lte*_. Moreover, this work also analyzes the underlying dynamical mechanism behind the above alpha rhythmic changes by nonlinear dynamical behavioral analysis. The results suggest that a decrease in synaptic connections *C*_*fte*_, *C*_*lfi*_, *C*_*pxe*_ and *C*_*tii*_ can promote transformation of the thalamic module from a limit cycle mode into a point attractor mode, which may be related with the slowing of alpha rhythm. The present results may have important implications, in particular, in understanding the neuronal correlates of alpha rhythm slowing associated with AD.

At last, we point out that in our modified TCT model there is a slowing of alpha rhythmic activity by decreasing some synaptic connectivity parameters associated with synapse loss in AD patients, which is consistent with the electrophysiological experimental result of EEG characteristics in AD patients. As indicated in the papers [45, 46], the EEG alpha rhythm is best seen with eyes closed and under the conditions of physical relaxation and mental inactivity. Yet the EEG alpha rhythm can be also blocked or attenuated by attentional shifts and mental effort, which is modulated by the thalamus and may be affected during pathological states like AD. This issue will be thoroughly investigated in our further work.

## Supporting information

S1 Dataset Data for the peak power density and the corresponding power spectral density with varying values of *C*_*fte*_. (ZIP)

S2 Dataset Data for the bifurcation analysis with varying values of *C*_*fte*_. (ZIP)

S3 Dataset Data for the phase orbits and the corresponding time series plots when *C*_*fte*_ takes different values. (ZIP)

S4 Dataset Data for the peak power density with varying values of *C*_*fte*_ for different values of *C*_*lte*_. (ZIP)

S5 Dataset Data for the peak power density and the corresponding power spectral density with varying values of *C*_*lfi*_. (ZIP)

S6 Dataset Data for the bifurcation analysis with varying values of *C*_*lfi*_. (ZIP)

S7 Dataset Data for the phase orbits and the corresponding time series plots when *C*_*lfi*_ takes different values. (ZIP)

S8 Dataset Data for the peak power density and the corresponding power spectral density with varying values of *C*_*pxe*_. (ZIP)

S9 Dataset Data for the bifurcation analysis with varying values of *C*_*pxe*_. (ZIP)

S10 Dataset Data for the phase orbits and the corresponding time series plots when *C*_*pxe*_ takes different values. (ZIP)

S11 Dataset Data for the peak power density and the corresponding power spectral density with varying values of *C*_*tii*_. (ZIP)

S12 Dataset Data for the bifurcation analysis with varying values of *C*_*tii*_. (ZIP)

S13 Dataset Data for the phase orbits and the corresponding time series plots when *C*_*tii*_ takes different values. (ZIP)
